# New High Affinity Monoclonal Antibodies Recognize Non-Overlapping Epitopes On Mesothelin For Monitoring And Treating Mesothelioma

**DOI:** 10.1038/srep09928

**Published:** 2015-05-21

**Authors:** Yi-Fan Zhang, Yen Phung, Wei Gao, Seiji Kawa, Raffit Hassan, Ira Pastan, Mitchell Ho

**Affiliations:** 1 Laboratory of Molecular Biology, Center for Cancer Research, National Cancer Institute, Bethesda, Bethesda, MD 20892, United States; 2 Thoracic and Gastrointestinal Oncology Branch, Center for Cancer Research, National Cancer Institute, Bethesda, MD 20892, United States

## Abstract

Mesothelin is an emerging cell surface target in mesothelioma and other solid tumors. Most antibody drug candidates recognize highly immunogenic Region I (296–390) on mesothelin. Here, we report a group of high-affinity non-Region I rabbit monoclonal antibodies. These antibodies do not compete for mesothelin binding with the immunotoxin SS1P that binds Region I of mesothelin. One pair of antibodies (YP218 and YP223) is suitable to detect soluble mesothelin in a sandwich ELISA with high sensitivity. The new assay can also be used to measure serum mesothelin concentration in mesothelioma patients, indicating its potential use for monitoring patients treated with current antibody therapies targeting Region I. The antibodies are highly specific and sensitive in immunostaining of mesothelioma. To explore their use in tumor therapy, we have generated the immunotoxins based on the Fv of these antibodies. One immunotoxin (YP218 Fv-PE38) exhibits potent anti-tumor cytotoxicity towards primary mesothelioma cell lines *in vitro* and an NCI-H226 xenograft tumor in mice. Furthermore, we have engineered a humanized YP218 Fv that retains full binding affinity for mesothelin-expressing cancer cells. In conclusion, with their unique binding properties, these antibodies may be promising candidates for monitoring and treating mesothelioma and other mesothelin-expressing cancers.

Mesothelin is a cell surface glycoprotein and tumor differentiation antigen highly expressed in many aggressive tumors such as mesothelioma, ovarian cancer, pancreatic adenocarcinomas, lung adenocarcinomas, and cholangiocarcinoma^1^,^2^,^3^,^4^. Thus, mesothelin is used as a serum and immunohistochemistry marker in cancer diagnosis^5^,^6^,^7^,^8^. Because it is shed from the cell^9^ and is present in biofluids such as serum, plasma, and pleural effusions, mesothelin can be detected via non-invasive approaches. These features are useful for cancer screening and for monitoring treatment response in cancers^7^,^8^. As a cell surface protein, mesothelin is also an emerging target for antibody therapeutics^10^,^11^,^12^,^13^,^14^. SS1P is an anti-mesothelin immunotoxin composed of an anti-mesothelin dsFv (SS1 Fv) fused to a 38 kDa *Pseudomonas* exotoxin-A fragment (PE38) and has been evaluated in clinical studies^12^,^14^. A recent study showed that SS1P in combination with pentostatin and cyclophosphamide resulted in major and prolonged tumor regressions in 3 of the 10 evaluable patients with malignant mesothelioma^14^. MORAb-009 (amatuximab), a chimeric anti-mesothelin monoclonal antibody (mAb) that contains the SS1 Fv for the same epitope, showed clinical activity as a single agent in a phase I trial^10^. Because the response to SS1P or MORAb-009 therapy, observed by radiographic studies, can take weeks to months to detect, it would be very useful to have a rapid blood test that is not interfered by the antibodies used for therapy. A plausible way to monitor early response to antibody treatment involves measuring the concentration of soluble mesothelin in biofluids. This can be achieved by a sandwich ELISA assay with one anti-mesothelin antibody coated plate to capture soluble mesothelin along with a second anti-mesothelin antibody to detect and quantify captured mesothelin^5^. However, a detection kit that measures mesothelin concentration in the presence of Region I binders such as MORAb-009 has not been reported because it is hard to make non-Region I antibodies.

Human mesothelin (MSLN) is a 40 kDa cell-surface glycosylphosphatidylinositol-linked glycoprotein (Fig. 1a). After being synthesized as a 71 kDa precursor and moved to the cell surface, the precursor is proteolytically processed and the 31 kDa amino terminus is removed as a megakaryocyte potentiating factor. The 40 kDa carboxyl terminus remains bound to the membrane as mature mesothelin and is referred to as mesothelin in this report^1^,^12^,^15^. MORAb-009 and SS1P recognize an epitope within the N-terminal Region I (296–390) of mesothelin^15^. However, Region I of mesothelin also interacts with other proteins which may interfere with the binding and function of anti-mesothelin region I antibodies. For example, MUC16/CA125, a protein that is often present in the serum of patients with mesothelin-related cancers, interacts with mesothelin^16^ via its Region I and competes with SS1^15^ and other Region I antibodies such as HN1, a human mAb^13^. To fully explore the potential of anti-mesothelin therapy and to search for antibodies that do not compete with the current therapeutic antibodies and their derivatives, we focused on the production of mAbs that reacted with the sub-domains of mesothelin that are distinct from the SS1 site recognized by SS1P and MORAb-009.

In the present study, we decided to make the antibodies that recognize previously undescribed epitopes on mesothelin beyond the SS1P/MORAb-009 site. To evaluate their potential in cancer diagnosis, we found that the antibodies were suitable for immunohistochemistry and they were highly sensitive for the detection of soluble mesothelin in ELISA. To explore their use in cancer treatment, we constructed immunotoxins based on new antibody Fvs and tested them *in vitro* and *in vivo*. An immunotoxin based YP218 was tested *in vivo* and showed good anti-tumor activity in a mesothelioma xenograft model in mice. In addition, we generated a humanized YP218 Fv that retained full binding affinity for mesothelin-expressing tumor cells. Based on these data, we believe that the mAbs described in this report have promising potential for cancer monitoring and treatment.

## Materials and Methods

### Immunization and rabbit mAb production

Rabbits were immunized with a rabbit Fc (rFc)-human mesothelin fusion protein^15^. Splenocytes from the immunized rabbits were isolated and fused with a rabbit plasmacytoma cell line as a hybridoma fusion partner^17^. Immunization of rabbits and fusions of hybridomas were performed by Epitomics (Burlingame, CA). Hybridomas were screened through combination of ELISA using mesothelin and its fragments^15^ and flow cytometry using Guava EasyCyte Plus instrument (Millipore, Billerica, MA)^18^. The rabbit mAbs were purified from culture supernatant on Protein A columns using an AKTA Explorer system (GE Healthcare, Fairfield, CT).

### Cell culture

The lung cancer cell line L55 and mesothelioma cell line M30 were kindly provided by Dr. Steven M. Albelda (University of Pennsylvania). The pancreatic cell line Panc3.014 was kindly provided by Dr. Elizabeth Jaffee (Johns Hopkins University). The detailed cell line information are summarized in Supplemental Table 1. All cells were incubated in 5% CO_2_ with the balance of air at 37 °C. Primary mesothelioma cells NCI-M-16, NCI-M-18, NCI-M-19, NCI-M-21 were cultured as previously described^19^. Hybridoma culture supernatants were collected, and positive wells were subcloned by limiting dilution in 96-well plates to obtain monoclonal lines. The antibody expression levels of these rabbit hybridomas as measured by ELISA are summarized in Supplemental Table 6.

### ELISA

**Figure 1:**
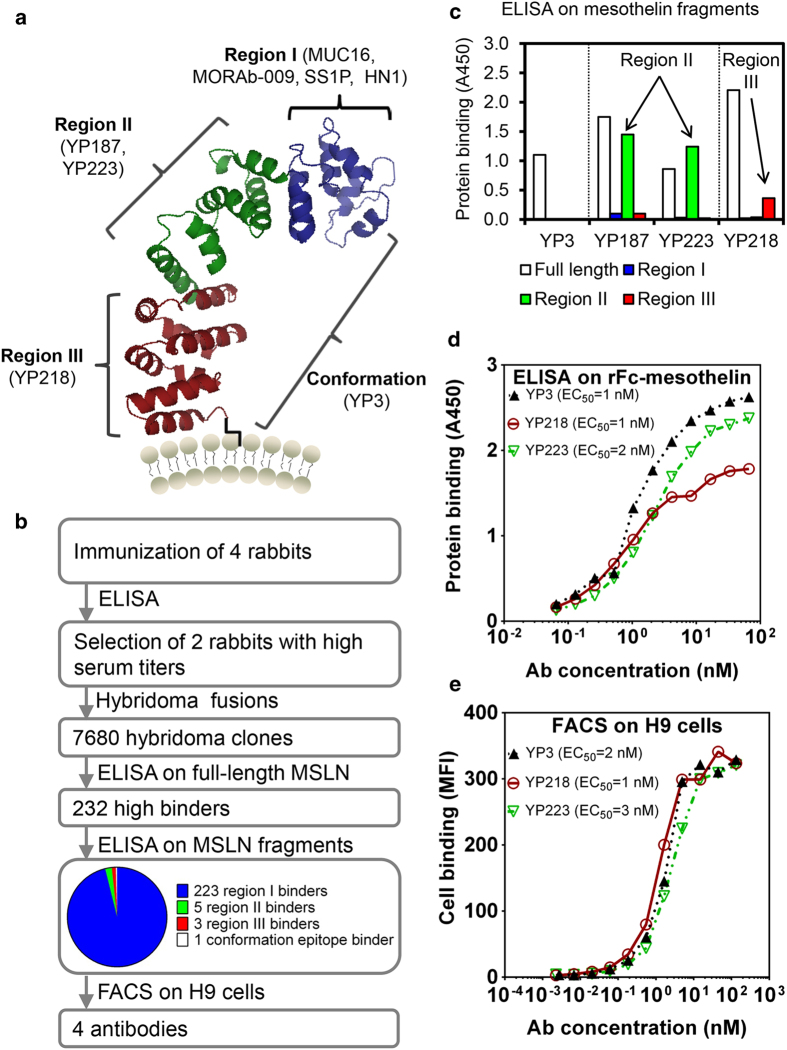
Generation and characterizations of rabbit antibodies to non-overlapping epitopes on human mesothelin. (**a**) A protein structure model of human mesothelin and the binding sites of new antibodies and current drug candidates. The protein structure model was built by I-TASSER software based on the mesothelin sequence (residues 296–598). (**b**) The procedure for screening rabbit antibodies to the epitopes distinct from the MORAb-009/SS1 site. (**c**) ELISA with mesothelin (MSLN) fragments (Regions I, II and III) and full-length MSLN. (**d**) Binding avidity (EC_50_) measurement of new antibodies by ELISA. E) Binding avidity (EC_50_) for mesothelin-expressing cells (H9). The binding signals were shown as mean fluorescence intensity (MFI) in flow cytometry.

To screen for domain-specific Abs, rabbit hybridoma supernatants were incubated with Nunc MaxiSorp 96-well flat-bottomed plates coated with 5 μg ml^−1^ of full-length mesothelin, Region I (residues 296–390), II (residues 391–486) or III (residues 487–598) fragments (Fig. 1a)^15^. Rabbit mAbs binding to mesothelin or its fragments were detected by a mouse anti-rabbit IgG light chain-specific HRP conjugate (Southern Biotech, Birmingham, AL). Visualization was achieved with 3,3′,5,5′-tetramethylbenzidine detection reagent (KPL, Gaithersburg, MD), and the absorbance was read at 450 nm with a SpectraMax Plus plate reader (Molecular Devices, Sunnyvale, CA). To determine the binding avidity of these rabbit mAbs, ELISA was performed in a similar method to that described above, except that plates were coated with 1 μg ml^−1^ of rFc-mesothelin fusion protein. The EC_50_ value was determined by Prism (version 3.02) for Windows (GraphPad software, San Diego, CA). As previously described^11^,^15^, EC_50_ was calculated based on the concentration needed to achieve a half-maximum binding.

In order to confirm that binding of selected rabbit Abs did not compete with the SS1P binding site, we performed several sandwich ELISA as described in the legend of Supplemental Figure 1.

To detect soluble mesothelin in the presence of SS1P, various concentrations of purified rFc-mesothelin fusion protein were mixed with or without SS1P (5 μg ml^−1^) in ELISA buffer (PBS with 0.01% Tween 20, 10% SuperBlock), followed by incubation with an ELISA plate coated with 5 μg ml^−1^ of YP223. Captured mesothelin was detected by biotinylated YP218 (5 μg ml^−1^, biotinylated with ChromaLink™ Biotin Antibody Labeling Kit from Solulink, San Diego, CA) and Streptavidin HRP conjugate (1:2000) (Invitrogen) sequentially. To detect soluble mesothelin in human serum, the same ELISA assay was used except that samples were diluted in PBST 1% BSA before measurement at 1:25 and 1:50, respectively. The effect of dilution factor on the calculated concentration of serum mesothelin was minor. The raw data were analyzed with Graphpad Prism (GraphPad Software, Inc., La Jolla, CA).

To detect soluble mesothelin in the presence of SS1P by MESOMARK^TM^ kit (Fujirebio Diagnostics Inc., Malvern, PA), we performed the ELISA using rFc-mesothelin with or without 5 μg ml^−1^ SS1P following manufacturer’s protocol. The rFc-mesothelin was used to calculate soluble mesothelin concentrations in the ELISA as previously described^6^.

### Flow cytometry

For screening of hybridomas, culture supernatant of rabbit hybridomas were incubated with suspended A431/H9 or A431 cells in a 96-well plate at 4 °C for 1 hour. Bound antibodies were labeled with Alexa Fluor 488 Rabbit IgG (Invitrogen). Cells were analyzed with Guava EasyCyte Plus (Millipore, Billerica, MA). To determine the binding affinity of rabbit antibodies, a similar protocol was used, except that the secondary antibody used was a goat anti-rabbit IgG-PE antibody (1:200) (Invitrogen). Cells were then analyzed using FACSCalibur (BD Biosciences, San Jose, CA) and the data were analyzed with FlowJo (Tree Star, Inc., Ashland, OR) and Graphpad Prism (GraphPad Software, Inc., La Jolla, CA).

To test the binding of different immunotoxins on the cell surface, serially diluted immunotoxins were incubated with different cell suspensions. The secondary antibody was rabbit anti-*Pseudomonas* Exotoxin A antibody (1:200) (Sigma-Aldrich, St. Louis, MO) and the third antibody was R-phycoerythrin Goat Anti-Rabbit IgG (H+L) (1:200) (Life Technologies, Grand Island, NY). Cells were analyzed with FACSCalibur, FlowJo and Graphpad Prism as described above. As described previously^18^ the EC_50_ value was calculated by Graphpad Prism. The surface expression level of mesothelin was measured as the binding of SS1P at 10 μg ml^−1^, which were quantified by comparison to the standard curve made with BD QUANTIBRITE™ PE BEADS (BD Biosciences, NJ, USA) according to the manufacturer’s protocol. The binding of immunotoxins at their IC_50_ were calculated based on the following formula: binding at IC_50_ = binding of SS1P at 10 μg ml^−1^ * log_10_ (fluorescence at IC_50_-fluorescence background)/log_10_ (fluorescence of SS1P at 10 μg ml^−1^-fluorescence background). We assume that one fluorophore corresponds to one antigen.

### Immunohistochemistry

Formalin-fixed paraffin embedded slides were deparaffinized in xylene and rehydrated through a graded ethanol series. Following an endogenous peroxidase block in 0.6% H_2_O_2_/methanol, antigen retrieval in 1mM EDTA pH 8.0 (10 min at 100 °C) was achieved in a Milestone RHS-1 Microwave Histoprocessor. After rinsing with PBS, sections were blocked with 2% normal goat serum (NGS) and incubated with primary antibodies (diluted in 0.1% BSA/PBS) at 4 °C overnight. The sections were then incubated with a biotinylated goat anti-rabbit IgG secondary antibody (diluted 1:100 in 1.5% NGS/PBS), stained with VECTASTAIN® ABC reagent (Vector Laboratories, Burlingame, CA) and developed with DAB (5 min). Slides were counterstained with hematoxylin. The primary Ab working concentrations were: 0.06 μg ml^−1^ YP3, <0.05 μg ml^−1^ Y187, 0.1 μg ml^−1^ YP218, 0.05 μg ml^−1^ YP223.

### Cloning of rabbit Ab Fv sequences

The rabbit Ab Fv sequences were cloned using 5’RACE. The primers (Supplemental Table 2) were designed based on the consensus nucleotide sequences of different allotypes of rabbit IgG CH1, hinge region, kappa C1 and kappa C2 in the IMGT database. To prepare cDNA templates, mRNA was extracted from hybridoma cells with Illustra^TM^ QuickPrep Micro mRNA Purification Kit (GE Healthcare, Buckinghamshire, UK). Five hundred nanograms of mRNA were reverse-transcribed into first strand cDNA with SuperScript TM III First-Strand Synthesis Supermix (Life Technologies, Grand Island, NY). The reaction mix was then treated with RNase H (NEB, Ipswich, MA) and processed for PCR purification (QIAquick PCR purification kit, Qiagen, Venlo, Netherlands). Poly-dC was added by terminal transferase (NEB, Ipswich, MA) to the 3’ end of the first strand cDNA (corresponding to the 5’ end of the mRNA). The products were purified with QIAquick PCR purification kit again before PCR reactions. The 5’RACE was performed using Phusion® Hot Start II High-Fidelity DNA Polymerase (Thermo Scientific, Cincinnati, OH). The Fv fragments were cloned into the pCR®4-TOPO® vector with TOPO® TA Cloning® Kit for Sequencing according to manufacturer’s instruction. Positive clones were screened with Taq DNA polymerase. BigDye (Invitrogen) was used for sequencing according to manufacturer’s protocol.

### Immunotoxin production and cytotoxicity measurement

Recombinant immunotoxin proteins were generated and their cytotoxicity as measured according to our published protocol^20^. The level of cell viability is measured with Cell Counting Kit-8 (Dojindo Molecular Technologies, Inc., Rockville, MD, USA) as WST (water-soluble tetrazolium salt) signals. IC_50_ values were measured by Graphpad Prism. To evaluate drug synergism, we serially diluted SS1P and YP218 Fv-PE38 starting from 1 μg/ml, incubated the two drugs in combination at different ratios and doses with the cells for three days, measured cell viability as described above, and used Compusyn (www.combosyn.com) to determine the combination index (CI) value. Using computerized simulations, synergism (CI < 1), additive effect (CI=1), and antagonism (CI > 1) were determined.

### Human subjects

Mesothelioma serum samples and primary tumor cells were obtained from patients with malignant mesothelioma. These patients were treated on an Institutional Review Board–approved protocol at the NCI (Bethesda, MD). Serum samples from healthy blood donors obtained from Bioreclamation (East Meadow, NY) were used as controls. All the experiments involving human subjects were carried out in accordance with the approved guidelines. Informed consent was obtained from all subjects.

### Animal testing

All mice were housed and treated under the protocol approved by the Institutional Animal Care and Use Committee at the National Institutes of Health (NIH). All the animal experiments were performed in accordance with the approved guidelines. A xenograft mouse model was made as previously described^21^. Five million LMB-H226-GL cells in 500 μl of PBS per mouse were injected intraperitoneally (i.p.) on Day 0. The SS1P (0.4 mg kg^−1^) or YP218 Fv-PE38 (0.4 mg kg^−1^) or PBS were injected i.p. on Day 12, 14, 16, 18. On Day 27 after injection with H226-GL cells, three mice were subjected to complete blood and serum chemistry testing for each treatment group (Supplemental Table 4). An additional three mice were dissected and their major organs weighed (Supplemental Table 5).

### Humanization of the YP218 Fv

The YP218 Fv was humanized via an approach adapted from a commonly used complementarity determining regions (CDR)-grafting method^22^. The VH and VL sequences were searched against the human germline sequence databases with IgBLAST (http://www.ncbi.nlm.nih.gov/igblast/) and IMGT/V-QUEST (http://www.imgt.org/IMGT_vquest/share/textes/). Based on this, the most similar human germ line Fv sequence and J region to YP218 were identified and used as a template. In the present study, we inserted both Kabat and IMGT CDR residues into the human germline framework.

## Results

### Generation of high affinity antibodies to various epitopes on human mesothelin

Our goal was to generate mAbs targeting previously undescribed epitopes in mesothelin for cancer diagnotics and therapy. To this end, we used rabbit hybridoma technology, because rabbit mAbs tend to exhibit more diverse specificities compared to conventional mouse mAbs^17^. We immunized four rabbits with rabbit Fc-mesothelin fusion protein^15^ expressed in human HEK-293 cells. Two rabbits whose sera showed the best binding to mesothelin in ELISA were selected for hybridoma fusions. Among the 7680 hybridoma clones, we chose 232 clones based on their high ELISA binding signals to mesothelin protein. We screened the 232 clones with an ELISA assay using a panel of mesothelin fragments generated in our lab, including Region I (residues 296–390), II (residues 391–486) and III (residues 487–598), and full-length mouse or human mesothelin^15^. We also screened these clones via flow cytometry analysis for live tumor cell binding. Among the 232 clones, 223 clones target Region I, 5 clones target Region II, 3 clones target Region III, and 1 clone is conformation sensitive as it only binds full-length mesothelin but not any mesothelin fragments. None of them bound mouse mesothelin (data not shown). Four clones were chosen for further study because of their unique domain binding patterns and strong cell binding signals (Fig. 1c). These clones included two mAbs binding region II (YP187 and YP223), one mAb binding region III (YP218), and one conformational epitope binder (YP3). The binding avidities of YP223, YP218, and YP3 to mesothelin were measured by both ELISA and Flow cytometry (Fig. 1d,e). EC_50_ values measured by the ELISA method were subnanomolar, within the avidity range of most approved therapeutic antibodies. The antibody expression levels of these rabbit hybridomas as measured by ELISA are summarized in Supplemental Table 6. In this study, we have successfully generated a group of mAbs that recognizes the epitopes in the regions distinct from the MORAb-009/SS1P site in human mesothelin.

### Potential application in tumor diagnosis

#### A new sandwich ELISA using YP218 and YP223 detect serum mesothelin with high sensitivity and specificity

**Figure 2:**
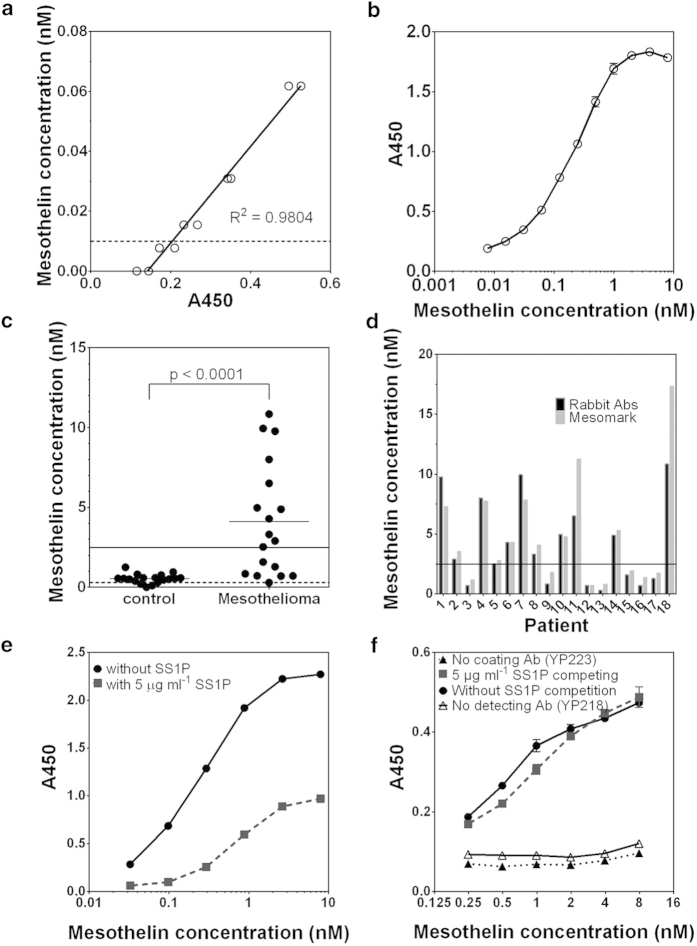
Detection of soluble mesothelin in human serum. (**a**) A new sandwich ELISA using YP218 and YP223. The detection limit is 0.01 nM (a dashed line). (**b**) The dynamic range of the assay. (**c**) Serum samples from patients with mesothelioma and healthy controls were tested for soluble mesothelin concentrations. The serum detection limit (0.25 nM) is indicated as a dashed line. The threshold between mesothelioma patient and healthy serum is indicated as a solid line^34^]. The mean values of each group were also shown. The difference between the two groups was statistically significant (*p* < 0.0001) using nonparametric Mann-Whitney test. (**d**) Comparison with the MESOMARK^TM^ assay. (**e**-**f**) SS1P inhibited the detection of soluble mesothelin by MESOMARK^TM^ (**e**) but not by the new assay (**f**). Error bars represent standard errors.

Serum mesothelin is a useful biomarker in malignant mesothelioma^7^,^23^. To determine whether the new antibodies can be used to detect soluble mesothelin, we conducted several sandwich ELISA assays (Supplemental Figure 1). Rabbit mAbs YP3, YP187, YP218, and YP223 bound wild-type mesothelin (both recombinant and secreted by mammalian cells in culture medium) in a dose-dependent manner in the presence of SS1P. Insterestingly, as shown in Supplemental Figure 1c-d, the conformation sensitive binder, YP3, bound wild-type mesothelin expressed by A431/H9 cells, but did not bind the mutant mesothelin with a 10-amino acid deletion (residues 411- VATLIDRFVK-420) in Region II expressed by A431/K5 cells, indicating that the conformation of the YP3 epitope is dependent on Region II of mesothelin. YP187 recognizing Region II of mesothelin also bound K5 weakly. YP218 and YP223 dose-dependently bound mutant mesothelin expressed by the K5 cell line. Based on these observations, we used YP218 and YP223 to establish a new sandwich ELISA to measure soluble mesothelin with the detection limit at 0.01 nM (Fig. 2a,b). The assay can be used to detect mesothelin in mesothelioma patient sera (Fig. 2c). Our new assay was comparable to the MESOMARK^TM^ kit in terms of sensitivity and specificity (Fig. 2d). We found that the average serum mesothelin level in mesothelioma patients was around 5 nM while its level in normal serum was below 2 nM. The serum mesothelin level was significantly elevated in mesothelioma patients as compared to normal healthy donors (*p* < 0.0001). Furthermore, SS1P inhibited the detection of soluble mesothelin by the MESOMARK^TM^ kit which presumably uses a Region-I mAb (Fig. 2e), we wondered whether the YP218 and YP223 mAbs can be used to measure soluble mesothelin in the presence of the SS1P immunotoxin. Indeed, we found SS1P did not affect measurement in our assay (Fig. 2f). Taken together, our results show that the new mAbs recognizing epitopes that are distinct from Region I should be useful to detect serum mesothelin in patients treated with MORAb-009/SS1P or similar region I antibodies.

#### New antibodies recognize mesothelin-positive human tumor tissues with high specificity

**Figure 3:**
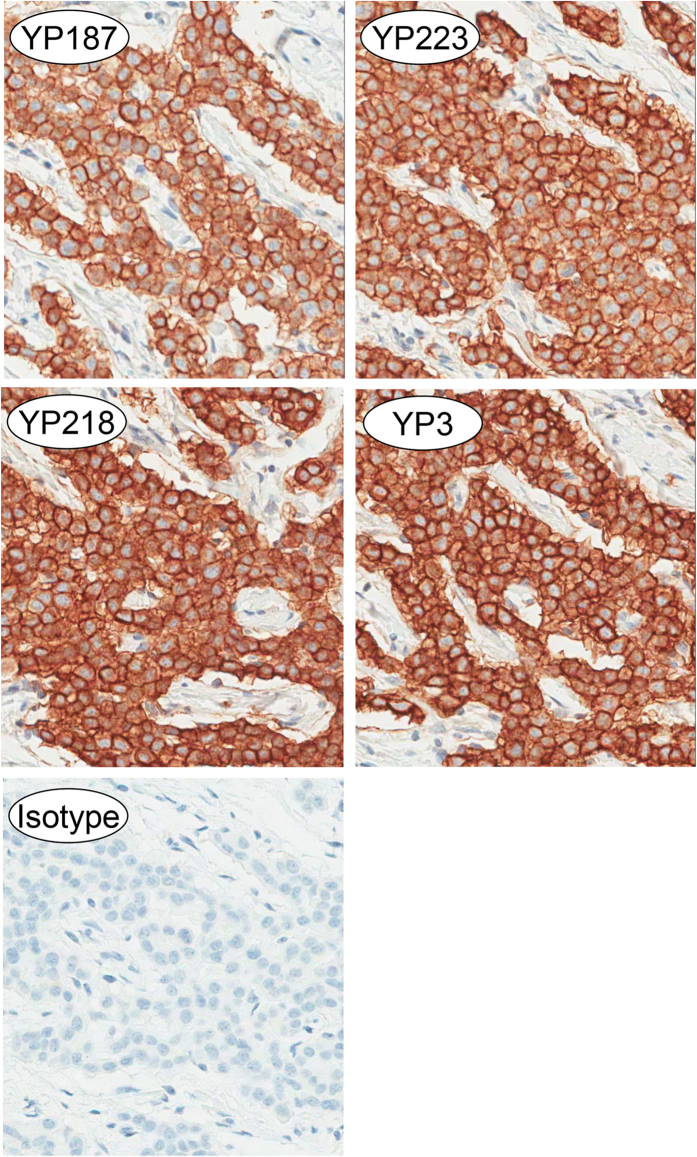
Immunohistochemistry with patient mesothelioma tissue sections stained with either an isotype control antibody or the anti-mesothelin antibody as indicated.

Mesothelin has been used as a tissue marker for mesothelioma and other cancers^7^. To evaluate potential use of the new mAbs in immunohistochemistry, fixed patient mesothelioma tissue was stained with YP187, YP223, YP218 and YP3 at an antibody concentration of 0.1 μg ml^−1^ or less (Fig. 3). The staining of tumor tissues was very strong and highly specific. The tumor cell membranes stained more strongly than the cytoplasm and there was no obvious staining in the nucleus. Collectively, these new antibodies are highly specific for tumor tissues, indicating that they are suitable for immunodiagnostics.

### Generation of anti-mesothelin immunotoxins for cancer treatment

**Figure 4:**
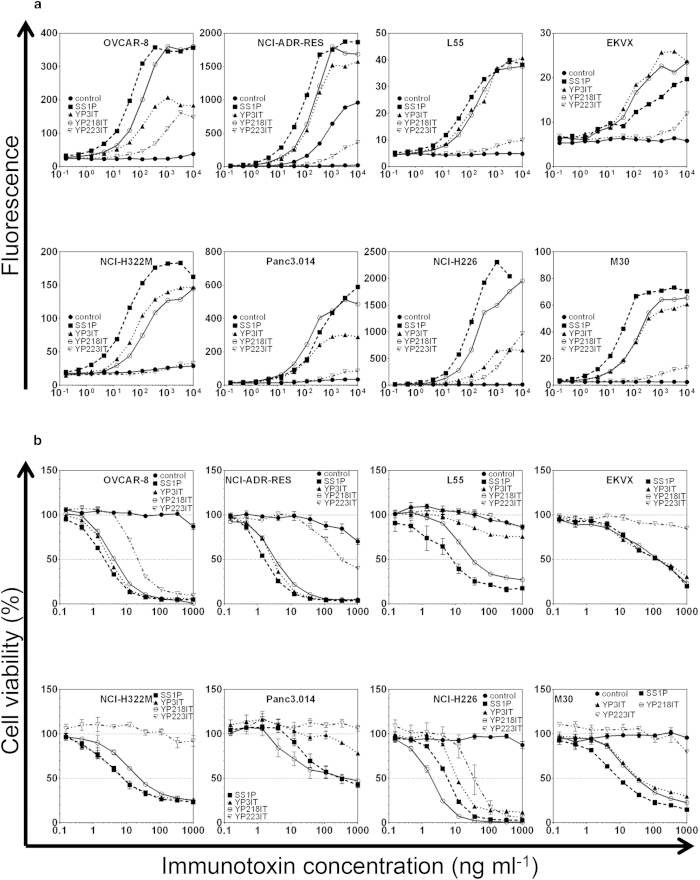
Construction and characterization of anti-mesothelin immunotoxins. (**a**) Live cell binding analysis of anti-mesothelin Fv-PE38 (immunotoxins) via flow cytometry. The functional binding affinity was summarized in Supplemental Table 3. The order of binding affinity varies between different cell lines. (**b**) Cytotoxicity of anti-mesothelin Fv-PE38 (immunotoxins) *in vitro*. The order of cytotoxicity varies between different cell lines. The WST signal measures dehydrogenase activities, which is directly proportional to the number of living cells. Error bars indicate standard errors. IT, immunotoxin.

**Figure 5:**
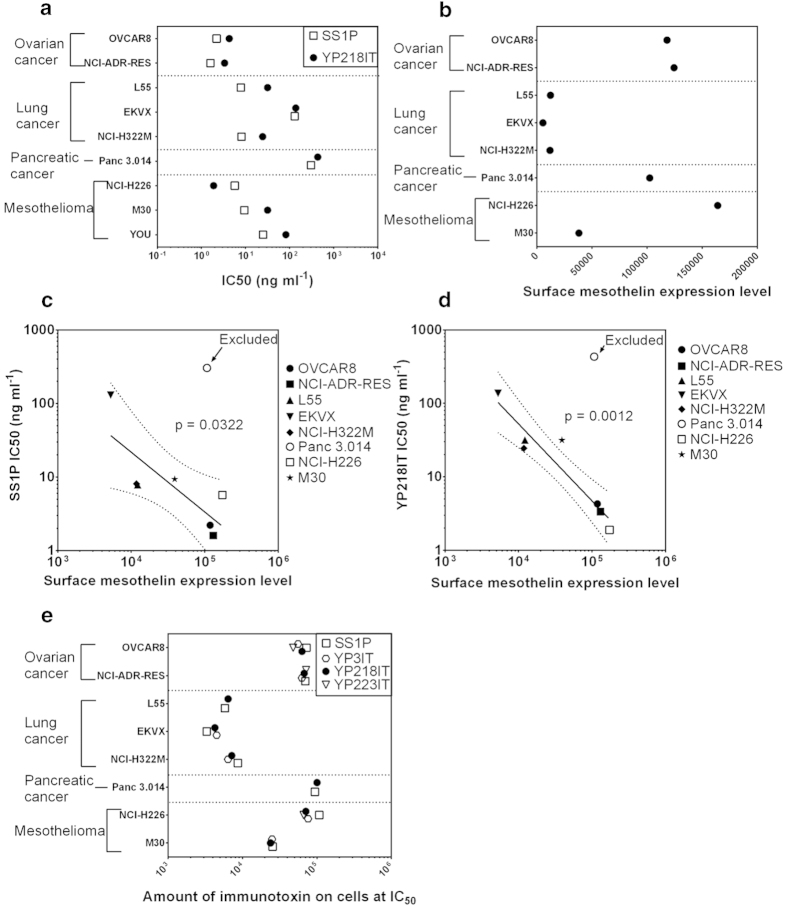
The comparison of affinity and cytotoxicity of anti-mesothelin immunotoxins on various solid tumor cell lines. (**a**) The IC_50_ of anti-mesothelin immunotoxins on various solid tumor cell lines. (**b**) The surface mesothelin expression level of various solid tumor cell lines. (**c-d**) The IC_50_ and surface mesothelin expression levels were inversely correlated for most of the tested cell lines except the outlier, Panc 3.014, which was excluded from the statistical analysis. The inverse correlations were significant for both SS1P (**c**) and YP218 Fv-PE38 (**d**). The dotted line shows the 95% confidence intervals of the regression slope. The p values were calculated with the linear regression function by Graphpad Prism. (**e**) The amount of binding required to kill 50% of the same cell type was similar among different immunotoxins on most of the cell lines. The amount of immunotoxin binding at IC_50_ concentrations were calculated by comparing the binding curve to the standard curve made by BD QUANTIBRITE™ PE BEADS. IT, immunotoxin.

To determine whether the new antibodies can be used as therapeutic candidates, we constructed immunotoxins. We produced similar single chain Fv-PE38 fusion proteins for the rabbit Abs and compared them with the SS1P immunotoxin. We first compared the cytotoxicity and affinity of YP3 Fv-PE38, YP218 Fv-PE38, YP223 Fv-PE38 and SS1P *in vitro* with several solid tumor cell lines (Figs. 4,5a, Supplemental Table 3). The immunotoxins have potent anti-tumor activity on ovarian cancer (OVCAR8 and NCI-ADR-RES), lung adenocarcinomas (L55, EKVX and NCI-H322M) and mesothelioma (NCI-H226 and M30) cells. The efficacy of these immunotoxins may be possibly influenced by the following factors: 1) surface mesothelin expression level, 2) immunotoxin affinity, 3) cell context and 4) immunotoxin binding epitope. To analyze the immunotoxins, we quantitatively measured the surface mesothelin expression level for these cell lines by flow cytometry (Fig. 5b). We found that the IC_50_s of SS1P and YP218 Fv-PE38 were inversely correlated with the mesothelin surface expression level, if the outlier, Panc 3.014, was excluded from the analysis (Fig. 5c,d). Pancreatic cancer cells (e.g., Panc 3.014) were very resistant to anti-mesothelin immunotoxins as previously described^24^,^25^.

**Figure 6:**
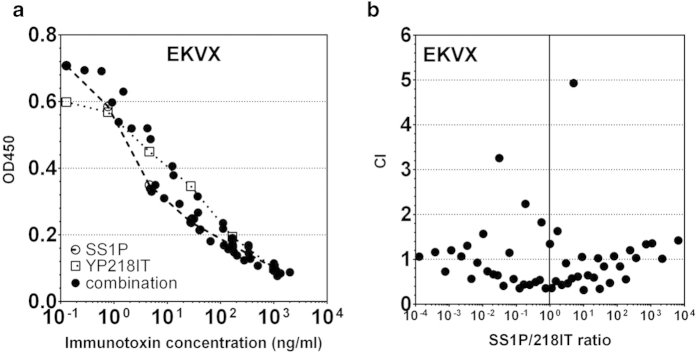
The cytotoxicity of the combination of SS1P and YP218 immunotoxins compared to the cytotoxicity of single reagents. (**a**) Different ratios of combinations were plotted together with the dose-response curve of single drugs. (**b**) CI values were calculated by Compusyn and plotted against the ratio of SS1P and YP218 immunotoxins. The CI value clustered around 1. IT, immunotoxin.

**Figure 7:**
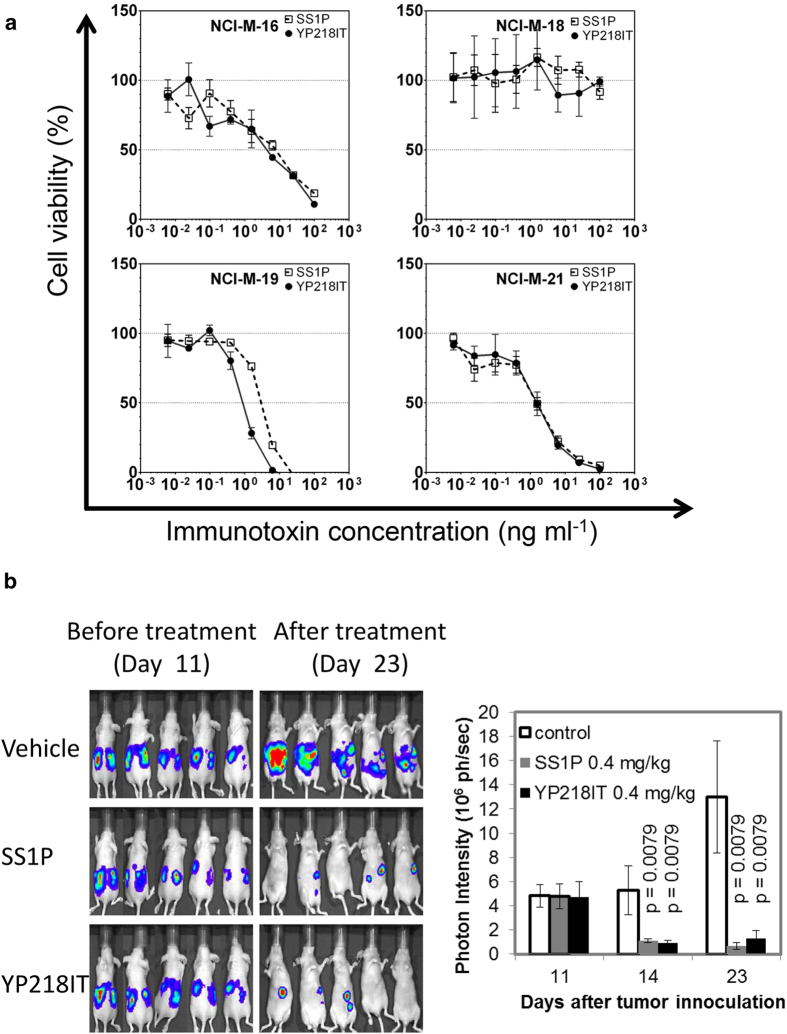
The cytotoxicity of YP218 Fv-PE38 in primary cell lines from mesothelioma patients and in an NCI-H226 orthotopic mesothelioma xenograft model. (**a**) Cytotoxicity of YP218 Fv-PE38 and SS1P in primary cell lines from patients. (**b**) Anti-tumor activity of YP218 Fv-PE38 and SS1P in a mouse xenograft model with NCI-H226. Error bars indicate standard errors. The *p* values were calculated by comparing with controls in nonparametric Mann-Whitney test. Tumor cells were injected on Day 0. IT, immunotoxin.

We also found that the immunotoxin with higher affinity generally had a stronger cytotoxicity (Supplemental Table 3, *p* < 0.0001 in Spearman’s rank correlation test). As shown in Fig. 5e, at its IC_50_ concentration, the amount of surface-bound immunotoxin molecules were similar among various immunotoixns for each cell line although they bind different epitopes. Therefore, the different anti-tumor activities (IC_50_s) of different immunotoxins may be attributed to their different binding affinities for mesothelin instead of their different epitopes. If we assume that each fluorophore corresponds to one antigen, then 10^4^–10^5^ immunotoxin molecules per cell are necessary and sufficient to kill 50% of target tumor cells. Interestingly, while ovarian cancer cells (OVCAR8 and NCI-ADR-RES) and mesothelioma (NCI-H226) require 10^5^ immunotoxin molecules to achieve its IC_50_, lung adenocarcinoma cells (L55 and NCI-H322M) need fewer immunotoxin molecules (10^4^). The combination of SS1P and YP218 immunotoxins showed an additive effect and no obvious synergistic effect in EKVX (a cell line with low mesothelin expression levels) (Fig. 6). In addition to exhibiting slightly more cytotoxicity than SS1P in the NCI-H226 human mesothelioma line (Fig. 4b), YP218 Fv-PE38 also showed higher affinity than other rabbit anti-mesothelin immunotoxins on most of the cell lines (Supplemental Table 3). In light of this, we decided to further compare the cytotoxicity of YP218 Fv-PE38 and SS1P *in vitro* with four primary cell lines isolated from malignant mesothelioma patients (Fig. 7a, Supplemental Table 3). We found that YP218 Fv-PE38 showed greater cytotoxicity than SS1P in NCI-M-19 and comparable cytotoxicity in NCI-M-16 and NCI-M-21, whereas NCI-M-18 was resistant to both immunotoxins.

To further analyze its potential value in cancer therapy, we tested YP218 Fv-PE38 in a mouse xenograft model with the NCI-H226 line expressing GFP and luciferase (LMB-H226-GL). This xenograft model allowed us to visualize tumor growth quantitatively^21^. SS1P and YP218 Fv-PE38 were used at the same dosage (0.4 mg kg^−1^). The mice were treated with immunotoxins every other day for four times. Both SS1P and YP218 Fv-PE38 shrank the tumor and their effect was similar (Fig. 7b). At the end of the experiment, we performed *in vivo* toxicology studies. Most serum chemistry and blood cell counts in YP218 Fv-PE38 treated group were similar to those of the untreated group and SS1P-treated group (Supplemental Table 4). Most of the parameters for liver, kidney and pancreas functions were within the normal range in all groups of mice. All groups of mice had similar weight of brain, heart, kidney, liver, lung and spleen (Supplemental Table 5). These data demonstrated that the YP218 Fv-PE38 immuntoxin had no severe *in vivo* toxicity in mice. Taken together, the non-Region I antibodies are promising candidates as cancer therapeutics.

#### Humanization of rabbit Fv YP218

**Figure 8:**
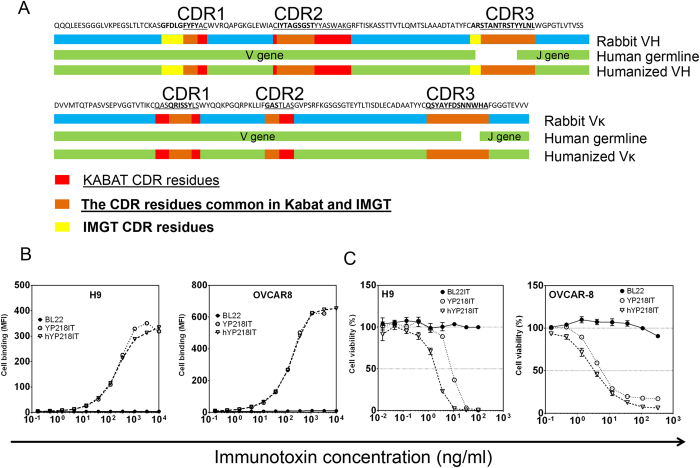
Humanization of the YP218 antibody. (**a**) Schematic view of CDR grafting. The CDR regions are defined according to Kabat (underlined) or IMGT (boldface) numbering. (**b**) The binding of hYP218 immunotoxin and YP218 immunotoxin on tumor cells. (**c**) The cytotoxicity of hYP218 immunotoxin and YP218 immunotoxin. IT, immunotoxin.

To further explore the therapeutic value of YP218, we humanized the rabbit Fv via a modified CDR grafting method without back-mutation. The template was based on the most similar human germline sequences to YP218. More specifically, these were IGHV3-23*05 for VH grafting and IGKV1-27*01 for VL grafting (Fig. 8a). We inserted both Kabat and IMGT CAR residues into the human antibody germline framework. The resulting hYP218 immunotoxin retained full binding affinity for mesothelin (Fig. 8b), and the cytotoxicity slightly increased (Fig. 8c). Although there was an extra cysteine at position 80 (Kabat numbering) in the rabbit VL sequence, after replacing it with proline in the human framework sequence, we did not notice any change in the loss of binding for the antigen. Therefore, we have successfully humanized the YP218 rabbit Fv for potential clinical applications in humans.

## Discussion

In this study, we generated a new group of rabbit mAbs specific for previously undescribed epitopes in mesothelin, and showed that 1) the new antibodies such as YP218 and YP223 can be used to detect soluble mesothelin in the presence of Region I binders including SS1P, 2) they are suitable for immunohistochemistry, 3) the immunotoxin based on YP218 exhibits potent anti-tumor activity in mice bearing xenograft mesothelioma and has no severe *in vivo* toxicitiy, and 4) the rabbit YP218 Fv can be humanized by CDR grafting without loss of binding affinity for the antigen. These observations make new mAbs promising candidates for monitoring and treating mesothelioma and other mesothelin-expressing tumors. This is one of the few reports where rabbit monoclonal antibodies are explored for therapeutic development.

The rabbit has long been used as a source of polyclonal antibodies. Not only are they able to develop antibodies that recognize diverse epitopes (including post-translational modifications such as glycosylation^26^ and phosphorylations), rabbits are evolutionarily distant from mice and may recognize epitopes distinct from mice and human^27^. In recent years, rabbit monoclonal antibodies have been isolated by phage display or hybridoma technology^27^,^28^,^29^. In the present study, we generated 7680 hybridoma clones from two rabbits, much more than the number of clones generated by mouse hybridoma technology (e.g. ~1000 clones from two mice^18^). The number of mesothelin-specific clones was much higher in rabbits immunized with rabbit Fc (rFc)- mesothelin fusion protein (232 clones from two rabbits) compared to mesothelin-deficient mice immunized with full-length-mesothelin DNA and boosted with rFc- mesothelin fusion protein (only 17 clones from two mice)^30^. Our study showed that a vast majority of anti-mesothelin rabbit antibodies (223 of 232 clones) recognized Region I. In mesothelin-deficient mice, 15 of the 17 anti-mesothelin mouse antibodies bind a similar epitope as SS1 and K1, which recognize region I, whereas the other two bind an unknown epitope^30^. This indicates that in both rabbit and mouse, region I of mesothelin is the most immunogenic portion. Anti-mesothelin autoantibodies have been detected in patients with mesothelioma and ovarian cancer^31^,^32^. Since a majority of autoantibodies target Region I, it may be advantageous to have antibodies targeting regions distinct from Region I in mesothelin.

There may potentially be many hybridomas that recognize conformation epitopes because we immunized the rabbits with full-length mesothelin protein, but, interestingly, only one (YP3) was identified to be conformation sensitive. We identified that YP3 binding is dependent on a 10-amino acid sequence in Region II of mesothelin. Here, we also report the humanization of rabbit antibody YP218 by inserting both Kabat and IMGT CAR residues into the human antibody germline framework. Our method does not require back-mutations to optimize humanization. Unlike a previous report on rabbit antibody humanizations which inserted only Kabat CDR segments^29^, we included both Kabat and IMGT CDR segments in the humanization of rabbit antibodies. The resulting Fv (hYP218) has the same functional binding affinity for mesothelin-expressing tumor cells. An immunotoxin based on the hYP218 Fv had even stronger cytotoxicity than the original rabbit YP218-derived immunotoxin. The increase in cytotoxicity may be attributed to the highly stable human germline sequences (IGHV3-23*05 and IGKV1-27*01)^33^.

These antibodies may offer substantial potential value for tumor diagnosis. The combinatorial use of Abs targeting non-overlapping epitopes of mesothelin in sandwich ELISAs enabled us to measure the concentration of soluble mesothelin in the presence of SS1P, indicating its use for other Region I-based therapeutic agents (e.g. MORAb-009). We showed that the epitopes of YP218, YP223 and SS1P do not overlap each other. Importantly, a sandwich ELISA combining YP218 and YP223 brought the detection limit of soluble mesothelin (<0.01 nM) to 250-fold lower than the threshold between healthy people and malignant mesothelioma patients (>2.5 nM in serum^34^ and >20 nM in plueural effusions^35^). This detection limit is lower than that of MESOMARK^TM^ (0.16 nM)^36^], a commercial kit that has been approved to evaluate serum mesothelin in patients^37^. Clinical development will be needed to further validate our serum assay for monitoring cancer in patients. The new antibodies are also suitable for immunohistochemistry.

For potential cancer therapy, we tested immunotoxins targeting different epitopes, and showed that the cell surface mesothelin expression level and immunotoxin binding affinity were two major factors that determine the response of most cancer cell lines *in vitro*. As YP218 Fv-PE38 effectively shrank the NCI-H226 xenograft tumor and demonstrated potent anti-tumor activity in animal testing and primary cell lines, it may prove to be a promising candidate for cancer therapy.

The treatment of solid tumors is challenging, considering tumor response and treatment are significantly time-sensitive. Taking anywhere from several days to several weeks for signs of tumor shrinkage, traditional methods, including CT or MRI scan, are neither adequate nor timely enough. Moreover, some tumors, such as malignant pleural mesothelioma, have a diffuse pattern of growth and are difficult to image. Positron Emission Tomography with [18F] fluorodeoxyglucose can monitor these tumors, but is usually very costly and requires patients to fast for a minimum of eight hours prior to imaging^38^. A more convenient way to screen for the early response of mesothelin-expressing tumors is to monitor the concentration of mesothelin in biofluids. Given new developments highlighting the potential of anti-mesothelin immunotherapy with SS1P and MORAb-009, an ideal method will allow for the measurement of soluble mesothelin concentrations in the presence of these immunotherapeutic drugs. The sandwich ELISA assay described in our study can measure the concentration of soluble mesothelin at high sensitivity, even in the presence of Region I-based antibody drugs such as SS1P and MORAb-009. Overall, the new antibodies described here should be useful for emerging mesothelin-targeted cancer therapies and diagnostics.

## Author Contributions

IP and MH provided the original concept for the research and designed the study. YZ YP WG and SK performed the experiments, YZ YP RH IP and MH wrote the manuscript. All authors reviewed the manuscript.

## Additional Information

**How to cite this article**: Zhang, Y.-F. *et al.* New High Affinity Monoclonal Antibodies Recognize Non-Overlapping Epitopes on Mesothelin for Monitoring and Treating Mesothelioma. *Sci. Rep.* 5, 9928; doi: 10.1038/srep09928 (2015).

## Supplementary Material

Supporting Information
