# Supplementary material for: New High Affinity Monoclonal Antibodies Recognize Non-Overlapping Epitopes On Mesothelin For Monitoring And Treating Mesothelioma
Source: Sci Rep. 2015 May 21;5:9928. doi: 10.1038/srep09928 (PMC4440525; doi:10.1038/srep09928)
Supplement: Supporting Information [file srep09928-s1.pdf]

New High Affinity Monoclonal Antibodies Recognize Non-Overlapping Epitopes on Mesothelin for Monitoring and Treating Mesothelioma

Yi-Fan Zhang, Yen Phung, Wei Gao, Seiji Kawa, Raffit Hassan, Ira Pastan, Mitchell Ho

Supplemental Information:

Supplemental Figure 1. New antibodies recognize epitopes different from that of the SS1P site in ELISA. SS1P was used as a coating reagent and rabbit mAbs as detecting reagents. Rabbit Fc-mesothelin (**A**, **B**) or soluble mesothelin in the culture supernatant (**C**, **D**) was used as an antigen.

Supplemental Table 1. Cell lines and culture medium.

Supplemental Table 2. Primers used in Fv sequence cloning and confirmation.

Supplemental Table 3. Affinity (EC_{50}) and cytotoxicity (IC_{50}) of anti-mesothelin Fv-PE38 immunotoxins *in vitro*.

Supplemental Table 4. Complete blood counts and serum chemistry of mice carrying LMB-H226-GL xenograft. Blood samples were taken on Day 27 after tumor cell injection. Standard deviations are shown with the mean values.

Supplemental Table 5. Organ weight of mice carrying xenograft tumor that received immunotoxin treatment.

Supplemental Table 6. The expression levels of rabbit anti-mesothelin antibodies in hybridoma culture supernatant.

Supplemental Figure 1

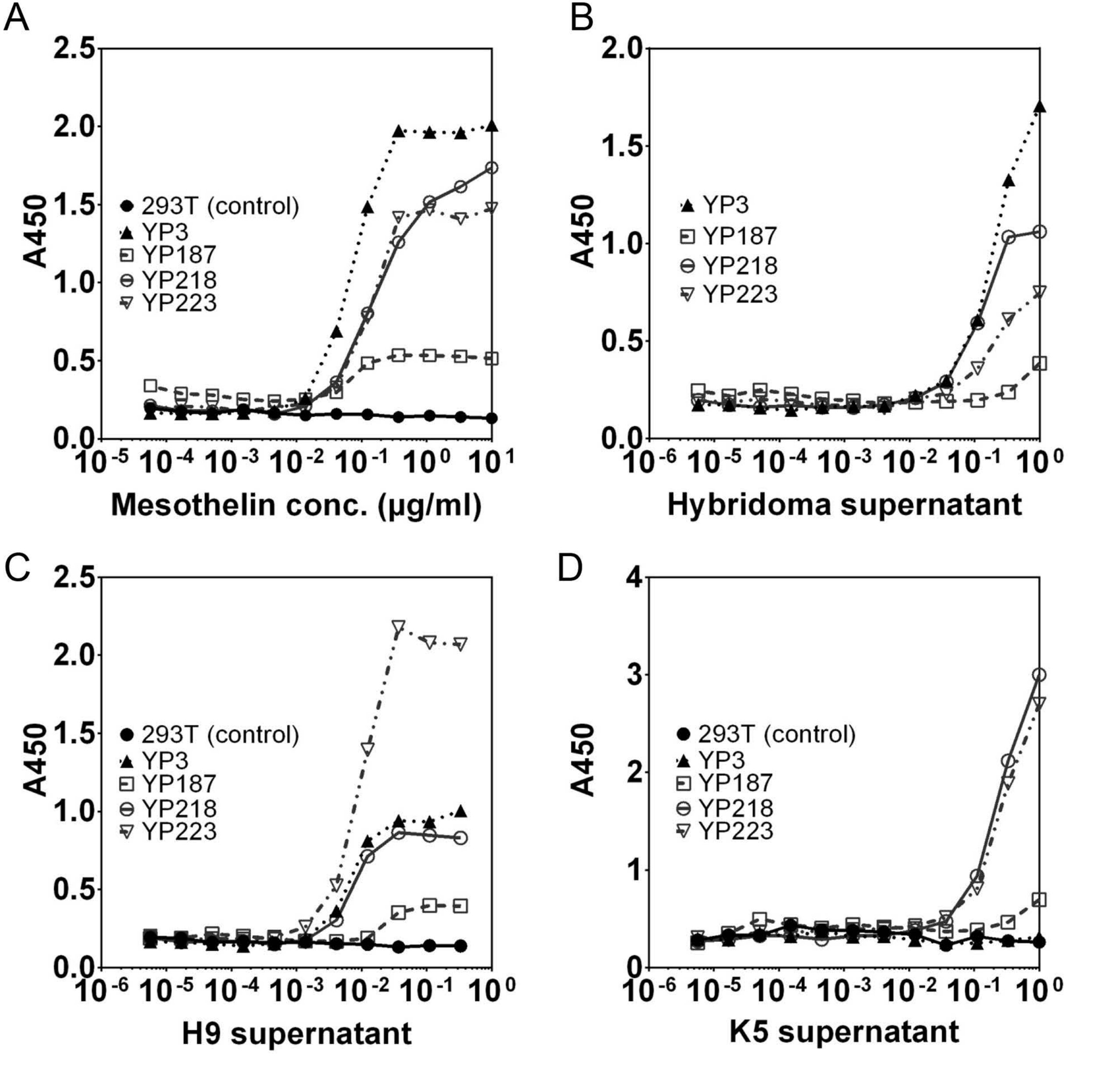

Supplemental Table 1. Cell lines and culture medium.

	culture medium		Source
**A431**	1	human epithelial carcinoma cell line	ATCC
**H9**	1	A431 that has been genetically engineered to overexpress full length wild type human mesothelin	Mitchell Ho and Ira Pastan (NCI, Bethesda, MD)
**K5**	1	A431 that has been genetically engineered to overexpress human mesothelin with 10-amino acid deletion (residues 411-VATLIDRFVK -420)	Ira Pastan (NCI, Bethesda, MD)
**OVCAR8**	2	Human metastatic ovarian cancer cell line	National Cancer Institute (NCI, Bethesda, MD; Development Therapeutics Program)
**NCI-ADR-RES**	2	Derived from OVCAR8	National Cancer Institute (NCI, Bethesda, MD; Development Therapeutics Program)
**L55**	2	human non-small cell lung adenocarcinoma cell line	Steven M. Albelda, (University of Pennsylvania, Philadelphia, PA)
**EKVX**	2	Human lung adenocarcinoma cell line	National Cancer Institute (NCI, Bethesda, MD; Development Therapeutics Program)
**NCI-H322M**	2	human bronchioalveolar (non-small cell) lung carcinoma cell line. Lung (cervical node metastasis) from human	National Cancer Institute (NCI, Bethesda, MD; Development Therapeutics Program)
**Panc 3.014**	2	Human pancreatic ductal adenocarcinoma cell line	Elizabeth Jaffee (John Hopkins University, Baltimore, MD)
**NCI-H226**	2	Human lung squamous non-small cell carcinoma, mesothelioma. Derived from metastatic site: pleural effusion	ATCC
**LMB-H226-GL**	2	Derived from NCI-H226	Mitchell Ho (NCI, Bethesda, MD)
**M30**	2	Human mesothelioma cell line	Steven M. Albelda, (University of Pennsylvania,

			Philadelphia, PA)
**YOU**	1	Human mesothelioma cell line	Raffit Hassan (NCI, Bethesda, MD)
**NCI-M-16 (passage 9)**	4	Human mesothelioma primary cell culture	Raffit Hassan (NCI, Bethesda, MD)
**NCI-M-18**	4	Human mesothelioma primary cell culture	Raffit Hassan (NCI, Bethesda, MD)
**NCI-M-19 (passage 10)**	4	Human mesothelioma primary cell culture	Raffit Hassan (NCI, Bethesda, MD)
**NCI-M-21 (passage 12)**	4	Human mesothelioma primary cell culture	Raffit Hassan (NCI, Bethesda, MD)
**YP223**	3	Rabbit hybridoma cell lines	Mitchell Ho (NCI, Bethesda, MD)
**YP218**	3	Rabbit hybridoma cell lines	Mitchell Ho (NCI, Bethesda, MD)
**YP3**	3	Rabbit hybridoma cell lines	Mitchell Ho (NCI, Bethesda, MD)
**YP187**	3	Rabbit hybridoma cell lines	Mitchell Ho (NCI, Bethesda, MD)

1. DMEM with 10% fetal bovine serum (FBS, from Hyclone, Thermo Scientific, Pittsburgh, PA), 1% L-glutamine, 1% penicillin, 1% streptomycin

2. RPMI 1640 (Invitrogen) supplemented with 10% FBS (Hyclone, Thermo Scientific, Pittsburgh, PA), 1% L-glutamine, 1% penicillin, and 1% streptomycin

3. RPMI 1640 supplemented with 10% FBS (Hyclone, Thermo Scientific, Pittsburgh, PA), RabMAb Supplement A (Epitomics), 0.05 mM 2-mercaptoethanol and 2 mM GlutaMAX-1 (Invitrogen)

4. RPMI 1640 (Invitrogen, Carlsbad, CA) supplemented with 20% FBS (Lonza, Walkersville, MD) 2 mM glutamine (Invitrogen), 100 units penicillin-streptomycin (Invitrogen) and 1 mM sodium pyruvate (Invitrogen)

Supplemental Table 2. Primers used in Fv sequence cloning and confirmation.

Primer's name	Sequence	Usage
rabbit IgG_hinge R	TGGGCTTGCTGCAYGTCGAGGG	RT* for IgG heavy chain
rabbit Ig CH1 285-262R	AACGGTCTTGTCCACTTTGGTGTT	1st PCR for IgG heavy chain
rabbit Ig CH1 196-177R	TCAGCGAGTAGAGGCCTGAG	2nd PCR for IgG heavy chain
rabbit Ig kappa C 312-292R	CCTRYTGAAGCTCTGGACGA	RT for Kappa chain
rabbit Ig kappa C1 219-194R1	CAGAGTgCTGCTGAGGTTGTAGGTA	1st PCR for Kappa chain
rabbit Ig kappa C1 219-194R2	CAGAGTaCTGCTGAGGTTGTAGGTA	1st PCR for Kappa chain
rabbit Ig kappa C2 219-194R	CAGAGTGCTGCTCAGGCTGTAGGTA	1st PCR for Kappa chain
rabbit Ig kappa C1&2 83-59R	CACACGATGGTGRCTGTTYCAGTTG	2nd PCR for Kappa chain
rabbit Ig lamda C 221-201R1	AGGCTGAGGAAGcTgCTGGCC	RT for Lamda chain
rabbit Ig lamda C 221-201R2	AGGCTGAGGAAGgTtCTGGCC	RT for Lamda chain
Adaptor+G	GGCCACGCGTCGACTAGTAC(G)10	1st PCR forward primer
Adaptor	GGCCACGCGTCGACTAGTAC	2nd PCR forward primer
RHFabVH1	GCTGCCCAACCAGCCATGGCCCAGTCGGTGGAGGAGTCCRGG	IgG VH forward primer for E. coli screening
RHFabVH2	GCTGCCCAACCAGCCATGGCCCAGTCGGTGAAGGAGTCCGAG	IgG VH forward primer for E. coli

		screening
RHFabVH3	GCTGCCCAACCAGCCATGGCCCAGTCGYTGGAGGAGTCCGGG	IgG VH forward primer for E. coli screening
RHFabVH4	GCTGCCCAACCAGCCATGGCCCAGSAGCAGCTGRTGGAGTCCGG	IgG VH forward primer for E. coli screening
rhfabvh5	CGATGGGCCCTTGGTGGAGGCTGARGAGAYGGTGACCAGGGTGCC	IgG VH reverse primer for E. coli screening
RHFabVk1	GGGCCCAGGCGGCCGAGCTCGTGMTGACCCAGACTCCA	VKappa forward primer for E. coli screening
RHFabVk2	GGGCCCAGGCGGCCGAGCTCGATMTGACCCAGACTCCA	VKappa forward primer for E. coli screening
rhfabvk3	AGATGGTGCAGCCACAGTTCGTTTGATTTCCACATTGGTGCC	VKappa reverse primer for E. coli screening
rhfabvk4	AGATGGTGCAGCCACAGTTCGTAGGATCTCCAGCTCGGTCCC	VKappa reverse primer for E. coli screening
rhfabvk5	AGATGGTGCAGCCACAGTTCGTTTGACSACCACCTCGGTCCC	VKappa reverse primer for E. coli screening
RHFabVlm1	GGGCCCAGGCGGCCGAGCTCGTGCTGACTCAGTCGCCCTC	VLamda forward primer
rhfabvlm2	AGATGGTGCAGCCACAGTTCGGCCTGTGACGGTCAGCTGGGTCCC	VLamda reverse primer

* RT: reverse transcription

Supplemental Table 3. Affinity (EC_{50}) and cytotoxicity (IC_{50}) of anti-mesothelin Fv-PE38 immunotoxins *in vitro*.

		Control	SS1P	YP3	YP218	YP223
**Ovarian cancer**						
OVCAR8	IC_{50} (ng/ml)	>1000	2	3	4	22
	EC_{50} (nM)		1	1	2	10
NCI-ADR-RES	IC_{50} (ng/ml)	>1000	2	3	3	320
	EC_{50} (nM)		1	3	3	23
**Lung cancer**						
L55	IC_{50} (ng/ml)	>1000	8	>1000	32	>1000
	EC_{50} (nM)		1	3	2	10
EKVX	IC_{50} (ng/ml)	>500	130	132	137	>1000
	EC_{50} (nM)		2	1	1	157
NCI-H322M	IC_{50} (ng/ml)	>300	8	8	24	>1000
	EC_{50} (nM)		0.4	1	2	9
**Pancreatic cancer**						
Panc 3.014	IC_{50} (ng/ml)	>1000	303	>1000	432	>1000
	EC_{50} (nM)		6	2	2	11

**Mesothelioma**						
NCI-H226	IC_{50} (ng/ml)	>1000	6	11	2	35
	EC_{50} (nM)		1	6	3	53
LMB-H226-GL	IC_{50} (ng/ml)		2	2	0.7	110
M30	IC_{50} (ng/ml)	>1000	9	35	32	>1000
	EC_{50} (nM)		0.5	2	2	12
YOU	IC_{50} (ng/ml)	>1000	25	49	82	>1000
	EC_{50} (nM)		2	4	7	28
**Primary mesothelioma**						
NCI-M-16	IC_{50} (ng/ml)		8		4	
NCI-M-18	IC_{50} (ng/ml)		>100		>100	
NCI-M-19	IC_{50} (ng/ml)		3		1	
NCI-M-21	IC_{50} (ng/ml)		2		2	
**other cell lines**						
A431 (mesothelin negative)	IC_{50} (ng/ml)	>100	>100	>100	>100	>100
A431/H9 (mesothelin+)	IC_{50} (ng/ml)	>1000	0.2	1	1	40
	EC_{50} (nM)		5	4	7	46

Supplemental Table 4. Complete blood counts and serum chemistry of mouse carrying LMB-H226-GL xenograft.

	Control	SS1P (0.4 mg/kg)	YP218 Fv-PE38 (0.4 mg/kg)	Normal Range
**White blood cells**				
White blood cells (K/μL)	7.1 ± 2.16	7.51 ± 0.97	14.37 ± 3.77	1.80 - 10.70
Neutrophils (K/μL)	2.04 ± 0.39	1.53 ± 0.30	3.19 ± 0.37	0.10 - 2.40
Lymphocytes (K/μL)	4.49 ± 1.83	5.71 ± 0.81	10.85 ± 3.54	0.90 - 9.30
Monocytes (K/μL)	0.43 ± 0.04	0.19 ± 0.10	0.21 ± 0.01	0.00 - 0.40
Eosinophils (K/μL)	0.12 ± 0.06	0.07 ± 0.02	0.1 ± 0.07	0.00 - 0.20
Basophils (K/μL)	0.023 ± 0.02	0.02 ± 0.01	0.02 ± 0.02	0.00 - 0.20
Neutrophils (%)	29.97 ± 8.04	20.32 ± 2.76	22.81 ± 3.59	6.60 - 38.90
Lymphocytes (%)	61.63 ± 9.93	76.07 ± 4.31	74.68 ± 4.85	55.80 - 91.60
Monocytes (%)	6.36 ± 1.53	2.52 ± 1.26	1.53 ± 0.47	0.00 - 7.50
Eosinophils (%)	1.72 ± 0.61	0.87 ± 0.16	0.81 ± 0.67	0.00 - 3.90
Basophils (%)	0.31 ± 0.12	0.22 ± 0.17	0.17 ± 0.17	0.00 - 2.00
Nucleated red blood cells (%)				RARE
**Erythrocytes**				
Red blood cells (M/μL)	9.47 ± 0.32	9.51 ± 0.02	10.06 ± 0.78	6.36 -

				9.42
Hemoglobin (g/dL)	14.53 ± 0.42	14.47 ± 0.65	15.13 ± 0.87	11.00 - 15.10
Hematocrit (%)	54.07 ± 0.67	54.67 ± 1.76	55.97 ± 4.06	35.10 - 45.40
Mean corpuscular volume (fL)	57.17 ± 2.31	57.47 ± 1.85	55.67 ± 0.40	45.40 - 60.30
Mean corpuscular hemoglobin (pg)	15.37 ± 0.65	15.2 ± 0.7	15.07 ± 0.35	14.10 - 19.30
Mean corpuscular hemoglobin concentration (g/dL)	26.87 ± 0.55	26.43 ± 0.67	27.07 ± 0.42	30.20 - 34.20
Red cell distribution width (%)	16.5 ± 0.46	16.17 ± 0.75	16.4 ± 0.4	12.40 - 27.00
**Thrombocytes**				
Platelets (K/µL)	692 ± 93.02	719.67 ± 85.24	804.33 ± 158.99	592.0 - 2972.0
Mean platelets volume (fL)	4.2 ± 0.17	4.3 ± 0.1	4.37 ± 0.31	5.00 - 20.00
**Serum**				
Albumin (g/dL)	4.4 ± 0.42	4.27 ± 0.42	4.3 ± 0.17	1.6 - 2.8
Alkaline phosphatase (U/L)	60.5 ± 9.19	54 ± 15.13	60.33 ± 3.79	67 - 282
Alanie aminotransferase (U/L)	61 ± 16.52	46.33 ± 4.51	51.33 ± 6.66	29 - 181
Amylase (U/L)	1357.33 ± 441.75	1435 ± 94.60	1484.33 ± 156.08	1691 - 3615
Total bilirubin (mg/dL)	0.3	0.3	0.4	0.0 - 0.6
Blood urea nitrogen (mg/dL)	25.67 ± 3.06	25 ± 2.65	23 ± 3.46	Dec-52
Ca (mg/dL)	11.63 ± 0.35	11.57 ± 0.42	11.7 ± 0.72	9.0 - 10.9

Phosphorus (mg/dL)	10.9 ± 0.42	12.57 ± 2.11	10.83 ± 0.57	5.5 - 12.4
Creatinine(mg/dL)	<0.2	< 0.2	< 0.2	0.2 - 0.4
Glucose (mg/dL)	263 ± 60.22	255 ± 58.13	261.33 ± 62.96	96 - 292
Na+ (mmol/L)	156.67 ± 3.21	158.67 ± 0.58	159.67 ± 3.21	145 - 158
K+ (mmol/L)	8.5 ± 0.1	9.25 ± 0.64	9.23 ± 0.47	5.5 - 8.7
Total protein (g/dL)	6.67 ± 0.64	6.47 ± 0.15	6.33 ± 0.75	4.2 - 5.9
Globulin (g/dL)	1.3 ± 1.13	2.17 ± 0.46	2.1 ± 0.87	0.0 - 0.6

The blood samples were taken on the 27th day after tumor cell injection. Standard deviations are shown with the mean values.

Supplemental Table 5. Organ weight of mouse carrying LMB-H226-GL xenograft.

	**Control**	**SS1P**	**YP218 Fv-PE38**
Brain	0.45 ± 0.04	0.43 ± 0.02	0.44 ± 0.06
Heart	0.12 ± 0.02	0.12 ± 0.01	0.13 ± 0.03
Kidney	0.31 ± 0.04	0.29 ± 0.02	0.31 ± 0.06
Liver	1.02 ± 0.29	1.01 ± 0.05	1.18 ± 0.21
Lung	0.16 ± 0.03	0.16 ± 0.04	0.17 ± 0.01
Spleen	0.14 ± 0.12	0.12 ± 0.02	0.14 ± 0.05

The organ weights were measured on the 27th day after tumor inoculation. The result was shown as mean ± standard deviation.

Supplemental Table 6. The expression levels of rabbit anti-mesothelin antibodies in hybridoma culture supernatant.

	Concentration in hybridoma supernatant (µg/ml)	Epitope
YP3	0.6	Conformational
YP187	0.17	Region II
YP223	1.2	Region II
YP218	1.5	Region III

Method:

1. Coat ELISA plate with goat anti-rabbit IgG Fc specific (Jackson ImmunoResearch 111-005-046) 5 µg/ml in 50 µl PBS/well, 4°C overnight.

2. Wash plate with PBS 0.05% Tween 20 (PBST) once.

3. Block plate at room temperature for 1 h with SuperBlock.

4. Add rabbit antibody standard (purified YP218) in PBST supplemented with 10% SuperBlock. Do 2 fold serial dilution starting from 2 µg/ml. At the same time, add culture supernatant of YP3, YP187, YP223, and YP218 supplemented with 0.05% Tween 20, serially diluted no more than 4 fold, 50 µl/well, room temperature 1h.

5. Wash plate with PBST for 3 times.

6. Add peroxidase conjugated goat anti-rabbit IgG (Jackson ImmunoResearch 111-035-046, 1/5000; or BioSource ALI3404, 1/2000) in 50 µl PBST/well, incubate at room temperature for 1 h.

7. Wash plate with PBST for 6 times.

8. Develop color.
